# Impact of nutrient excess on physiology and metabolism of *Sulfolobus acidocaldarius*

**DOI:** 10.3389/fmicb.2024.1475385

**Published:** 2024-10-04

**Authors:** Viktor Laurin Sedlmayr, Diana Széliová, Veerke De Kock, Yannick Gansemans, Filip Van Nieuwerburgh, Eveline Peeters, Julian Quehenberger, Jürgen Zanghellini, Oliver Spadiut

**Affiliations:** ^1^Research Division Biochemical Engineering, Institute of Chemical, Environmental and Bioscience Engineering, TU Wien, Vienna, Austria; ^2^Department of Analytical Chemistry, University of Vienna, Vienna, Austria; ^3^Research Group of Microbiology, Department of Bioengineering Sciences, Vrije Universiteit Brussel, Brussels, Belgium; ^4^Department of Pharmaceutics, Laboratory of Pharmaceutical Biotechnology, Ghent University, Ghent, Belgium

**Keywords:** *Sulfolobus acidocaldarius*, chemostat cultivation, overflow metabolism, carbon overfeeding, fatty acid metabolism, transcriptomic analysis, archaea, parsimonious flux balance analysis

## Abstract

Overflow metabolism is a well-known phenomenon that describes the seemingly wasteful and incomplete substrate oxidation by aerobic cells, such as yeasts, bacteria, and mammalian cells, even when conditions allow for total combustion via respiration. This cellular response, triggered by an excess of C-source, has not yet been investigated in archaea. In this study, we conducted chemostat cultivations to compare the metabolic and physiological states of the thermoacidophilic archaeon *Sulfolobus acidocaldarius* under three conditions, each with gradually increasing nutrient stress. Our results show that *S. acidocaldarius* has different capacities for the uptake of the two C-sources, monosodium glutamate and glucose. A saturated tricarboxylic acid cycle at elevated nutrient concentrations affects the cell’s ability to deplete its intermediates. This includes deploying additional cataplerotic pathways and the secretion of amino acids, notably valine, glycine, and alanine, while glucose is increasingly metabolized via glycogenesis. We did not observe the secretion of common fermentation products, like organic acids. Transcriptomic analysis indicated an upregulation of genes involved in fatty acid metabolism, suggesting the intracellular conservation of energy. Adapting respiratory enzymes under nutrient stress indicated high metabolic flexibility and robust regulatory mechanisms in this archaeon. This study enhances our fundamental understanding of the metabolism of *S. acidocaldarius*.

## 1 Introduction

Exposure to excessive nutrient concentrations can trigger remarkable responses in cells. In yeasts, such as *Saccharomyces cerevisiae*, high glucose availability results in less energy-efficient anaerobic combustion to ethanol, regardless of an oxygenated environment allowing for respiration. Likewise, bacteria, such as *Escherichia coli*, utilize the strategy of incomplete oxidation to acetate when a surplus of glucose is found in the environment, while lactate accumulation is associated with the cultivation of mammalian cells (Vazquez, 2018; Luginsland et al., 2024). This seemingly wasteful strategy results in less energy than complete oxidation via respiration; still, it occurs ubiquitously among fast-growing cells and is generally referred to as overflow metabolism. Although the exact reason for this phenomenon is still debated, it is widely accepted to result from a combination of several factors (Bernal et al., 2016). Szenk et al. argue that anaerobic combustion of C-sources is preferred in fast-growing cells due to an increased crowding of respiratory proteins caused by a reduced surface-to-volume ratio (Szenk et al., 2017). Co-cultivation experiments propose a competitive advantage of organisms exploiting an overflow metabolism by rapidly depleting nutrients and producing toxic metabolites, thereby inhibiting competing organisms (Yasir et al., 2024). Other theories suggest a protective measure against the harmful accumulation of metabolites (Li et al., 2022) or energy savings when employing fermentation (Basan et al., 2015).

Conversely, only little is known about the effect of nutrient surplus on archaea. Aerobic halophilic archaea, such as those in the genera *Haloferax* and *Haloarcula*, produce acetate as a byproduct during batch cultivation. Acetate is then consumed when the cells reach the stationary phase, similar to what occurs in *E. coli* (Oren and Gurevich, 1994; Kuprat et al., 2021). However, the mechanism of acetate formation in halophilic archaea differs significantly from their bacterial counterparts. *Haloferax* utilizes an ADP-forming acetyl-CoA synthetase to convert acetyl-CoA to acetate instead of the classical two-step reaction in bacteria (Bräsen and Schönheit, 2001). More detailed considerations regarding overflow metabolism are missing in the literature, particularly for the phylum Crenarchaeota. However, indications of incomplete glucose oxidation at high growth rates suggest the presence of overflow metabolism in crenarchaea (Rastädter et al., 2021).

*Sulfolobus acidocaldarius*, one of the best-characterized members of Crenarchaeota, thrives under extreme conditions, with an optimal pH = 3 and a growth temperature of 75 °C. *S. acidocaldarius* has been intensively investigated for several biotechnological applications, such as enzyme and lipid production (Quehenberger et al., 2017; Sedlmayr et al., 2024b). Only recently, the upscaling of a continuous cultivation process to 200 L was reported, solidifying the development toward industrial applications of extremophilic bioprocesses (Rastädter et al., 2023). Further progress in the biotechnological utilization of *S. acidocaldarius* requires progress in the basic understanding of metabolic behavior against stress factors to enhance process efficiency and product yields (Gecse et al., 2024). This knowledge can guide process intensification strategies, leading to improved yields, cost reductions, and enhanced sustainability in industrial processes. Apart from temperature and pH extremes, *S. acidocaldarius*’ natural habitat of acid thermal soils and acid hot springs is characterized by various environmental hurdles. Numerous studies have explored the extremophile’s responses to stress, revealing a versatile repertoire of metabolic, morphological, and physiological answers. Stress factors that have been studied include temperature (Baes et al., 2023), nutrient limitation (Bischof et al., 2019), solvent stress (Benninghoff et al., 2021), salt stress (Stracke et al., 2020), and pH stress (Buetti-Dinh et al., 2016).

In this study, chemostat cultivations were performed to assess the physiological and transcriptome response of *S. acidocaldarius* to excess nutrient concentrations. The defined Vienna Defined (VD) Medium, containing two C-sources, glucose and monosodium glutamate (MSG), was used as a substrate (Quehenberger et al., 2019). Chemostat cultivation is the direction the biopharmaceutical industry is heading, offering valuable advantages for experimental design (Kopp et al., 2019). It allows the cultivation at a precise specific growth rate and enables the manipulation of particular parameters with minimal impact on other process conditions (Mendes et al., 2013). By carefully adjusting the process parameters, we analyzed three conditions: (I) “Low,” with a moderate cell density and specific substrate uptake rate (q_s_), (II) “High,” with a higher cell density and q_s_, and (III) “Overfeed”: characterized by the highest cell density and q_s_, resulting in the accumulation of C-source in the broth. This experimental setup allowed us to differentiate between effects caused by high cell concentrations and impacts associated with excessive nutrient availability. With the help of RNA-sequencing (RNA-seq) and computational methods using parsimonious flux balance analysis, we obtained fundamental insights into the response of *S. acidocaldarius* to excessive nutrient availability, including shifts in energy and carbon management, respiration, and preferential use of different metabolic pathways.

## 2 Materials and methods

### 2.1 Strain and bioreactor setup

*S. acidocaldarius* DSM 639, obtained at the German Collection of Microorganisms and Cell Cultures (DSMZ, Germany), was grown continuously in a 2 L Biostat A-plus bioreactor (Sartorius, Germany), as described before (Rastädter et al., 2022). In short, the culture was stirred at 350 rpm, supplied with 0.25 vvm of pressurized air, and kept at a constant temperature of 75 °C. The pH was measured with an Easyferm Plus Electrode (Hamilton, USA) and controlled by the automatic addition of 4.8% H_2_SO_4_ at pH = 2.7. CO_2_ concentration in the exhaust gas was measured using a BCP-CO_2_ offgas analyzer (Bluesense, Switzerland). The cultivation was monitored and controlled using the Lucullus process control system (SecureCell, Switzerland).

### 2.2 Bioreactor cultivations

The batch phase was conducted within 1.5 L of VD Medium (Quehenberger et al., 2019) with a modified concentration of C-source (2 g/L MSG, 1 g/L glucose). During the fed-batch phase, an exponential feed was applied, starting with 16.5 g/h and a growth rate of 0.035 h^−1^. After reaching a working volume of 2 L, the chemostat phase was started by pumping cell broth out of the reactor via a bleed tube at a fixed height. Feed used during the chemostat phase consisted of a concentrated VD Medium with modified C-source concentrations (76 g/L MSG, 36 g/L glucose). An additional water feed was used to dilute the feed. The three different process conditions were set via differing the total flow rate (TFR) or the ratio between feed flow rate (FFR) and water flow rate (WFR). Samples for the condition “Low” derived from chemostat cultivation in the steady state at a TFR of 90 g/h, consisting of an SFR of 22.5 g/h and WFR of 67.5 g/h. Samples of the condition “High” were taken during steady state at a TFR of 120 g/h, composed of an SFR of 40 g/h and a WFR of 80 g/h. The same TFR was used for “Overfeed” samples, while the SFR was set to 60 g/h and the WFR to 60 g/h. After applying the Overfeed condition for 24 h, High settings were reapplied. Several transitions between Low and High were performed during the cultivation, where at least eight volumes exchanges were required to achieve a steady state. For successive samples of the same condition, at least three residence times were maintained between sampling. Two independent bioreactor cultivations were performed to generate five biological replicates per condition, which were used for transcriptome study and data evaluation.

### 2.3 Determination of biomass concentration via optical density and dry cell weight

Optical density (OD_600_) was determined photometrically on an ONDA V-10 PLUS (Giorgio Bormac, Italy) at 600 nm against a blank of deionized water. To determine dry cell weight (DCW), 2 mL of cell broth was transferred to tared 2 mL Eppendorf tubes and centrifuged (4 °C, 16,000×*g*, 10 min). The supernatant was discarded and the cell pellet dried in an oven at 105 °C. After 3 days, the mass of the cell pellet was measured on an analytical scale. OD_600_ was measured in duplicates, while DCW was determined in triplicates.

### 2.4 C-source and metabolite analysis

D-glucose-, pyroglutamate-, pyruvate-and trehalose content was analyzed in the clarified cell broth and the feed via HPLC measurement using an Aminex HPX-87H column (300 × 7.8 mm, Bio-Rad, United States) equipped with a pre-column (Micro-Guard Cation H+ cartridge, 30 × 4.6 mm; Bio-Rad, United States) using a Vanquish Core HPLC system (Thermo Fisher Scientific, United States). Pyruvate was analyzed via UV/vis detection at 210 nm. The remaining analytes were detected by measurements with a refractive index detector (RefractoMax 520, IDEX Health & Science, United States). The column temperature was 60 °C, while the flow rate was 0.6 mL/min. Isocratic elution was achieved with 4 mM H_2_SO_4_. Chromatograms were analyzed using Chromeleon 7.2.10 ES Chromatography Data System (Thermo Fisher Scientific, United States). For quantification, seven standard solutions were prepared for glucose (10 g/L − 0.156 g/L), pyroglutamate (1 g/L − 0.0156 g/L), pyruvate (2 g/L − 0.0313 g/L) and trehalose (2 g/L − 0.0625 g/L) by serial dilution. Glutamate was analyzed photometrically with a Cedex Bio HT Analyzer (Roche, Switzerland).

### 2.5 Amino acid analysis

Liquid Chromatography-Electrospray Ionization-Mass Spectrometry (LC-ESI-MS) was performed to quantify secreted amino acids in the supernatant. Before analysis, samples and standards were diluted using 80% acetonitrile. For each sample, four dilutions were prepared (1:4, 1:10, 1:100, 1:1,000). The calibration curves for every amino acid were done using a commercial amino acid standard (AAS18, amino acid standard solution), which was performed for a serial dilution ranging from 52.1 μM to 0.104 μM. Standards and samples were analyzed on an iHILIC P classic column (2.1*100 mm, 5 μm; HILICON, Sweden) using 95% water, 5% acetonitrile, 20 mM NH_4_HCO_3_, 0.1% NH_4_OH, 2.5 μM medronic acid as solvent (A) and 95% acetonitrile, 5% water, 2.5 μM medronic acid as solvent (B). The total analysis time was 22 min. A 250 μL/min flow rate was sustained from 0 to 15.5 min and 20.5–22 min. Between 16.5 and 20 min, the flow rate was raised to 400 μL/min. After 1 min of 90% (B), an 11-min gradient was implemented, gradually reaching 35% (B). Within 0.5 min, (B) was reduced to 25% and maintained for 2 min. Subsequently, within another 0.5 min, solvent (B) was again increased to 90% and maintained for 7 min. Column oven was set to 40 °C. The amino acids were detected with a triple quadrupole (TSQ Vantage, Thermo Scientific, United States) equipped with an H-ESI source in positive ion mode. Data evaluation was performed in Skyline 23.1.0.268. The limit of quantification for the individual amino acids can be found in Supplementary Table S1.

### 2.6 Calculation of rates and biomass yield

*Specific growth rate*, μ [1/h], at time point t_n_, was determined as the difference in DCW between the sampling point t_n_ and the previous sampling point t_n-1_ divided by the average DCW between the sampling points times the time difference between the sampling points, considering the cumulative loss of biomass via the bleed (Equation 1).

Calculation of the specific growth rate:


(1) $$\mu =\frac{\left(x_{t_{n}}-x_{t_{n-1}}\right)*V_{\mathrm{reactor}}+\bar{x\;}*\Delta V_{\mathrm{Bleed}}}{\bar{x\;}*V_{\mathrm{reactor}}*\left(t_{n}-t_{n-1}\right)}$$

- t_n_ [h] = sampling time point n
- t_n-1_ [h] = sampling time point before n
- x_tn_ [g/L] = volumetric DCW at t_n_
- x_tn-1_ [g/L] = volumetric DCW at t_n-1_
- V_reactor_ = reactor volume
-
$\bar{x}$ [g/L] = average volumetric DCW between t_n_ and t_n-1_
- ΔV_Bleed_ [L] = bleed volume removed between the two sampling points

*Specific substrate uptake rates* for glucose, q_Glc_ [g_Glc_/g_x_/h], and MSG, q_MSG_ [g_MSG_/g_x_/h], between two sampling points, were calculated as the difference of the absolute substrate content in the broth plus the absolute substrate content that was supplied to the reactor minus the absolute substrate content that was discharged in that period divided by the average DCW between those two sampling points times the time difference between the sampling points (Equation 2). The overall specific substrate uptake rate q_s_ [g_s_/g_x_/h] was calculated as the sum of q_Glc_ and q_MSG_.

Calculation of the specific substrate uptake rate:


(2) $$q_{S}=\frac{\left(s_{t_{n}}-s_{t_{n-1}}\right)*V_{\mathrm{reactor}}+s_{\mathrm{in}}*V_{\mathrm{in}}-\bar{s\;}*V_{\mathrm{out}}}{\bar{x\;}*V_{\mathrm{reactor}}*\left(t_{n}-t_{n-1}\right)}$$

- s_tn_ [g/L] = substrate concentration in broth at t_n_
- s_tn-1_ [g/L] = substrate concentration in broth at t_n-1_
- s_in_ [g/L] = substrate concentration in feed
- V_in_ [L] = volume supplied to bioreactor between t_n_ and t_n-1_
- s̄ [g/L] = average substrate concentration between t_n_ and t_n-1_
- V_out_ [L] = volume discharged from bioreactor between t_n_ and t_n-1_

*Specific production rates* of trehalose, q_Trehalose_ [g_Trehalose_/g_x_/h], and of the individual amino acids, q_AA_ [g_AA_/g_x_/h], (where the subscript “AA” denotes the specific production rate of each individual amino acid) between two different sampling time points, were calculated as the absolute mass of product formed in the bioreactor in that period minus the absolute product that was discharged in that period (Equation 3).

Calculation of the specific production rate:


(3) $$q_{P}=\frac{\left(p_{t_{n}}-p_{t_{n-1}}\right)*V_{\mathrm{reactor}}+p_{\mathrm{in}}-p_{\mathrm{out}}}{\bar{x\;}*V_{\mathrm{reactor}}*\left(t_{n}-t_{n-1}\right)}$$

- p_tn_ [g/L] = product concentration in broth at t_n_
- p_tn-1_ [g/L] = product concentration in broth at t_n-1_
- p_in_ [g/L] = product concentration in feed
- p_out_ [g/L] = product concentration in bleed
- p̄ [g/L] = average product concentration between t_n_ and t_n-1_

*Substrate accumulation rate* [g/L/h] between two sampling points was calculated using Equation 4.

Calculation of the substrate accumulation rate:


(4) $$\mathrm{accumulation rate}=\frac{s_{t_{n}}-s_{t_{n-1}}}{t_{n}-t_{n-1}}$$

*Substrate uptake rates* for glucose, r_Glc_ [g_Glc_/h], and MSG, r_MSG_ [g_MSG_/h], between two sampling points, were calculated as the difference of the absolute substrate content in the broth plus the absolute substrate content that was supplied to the reactor minus the absolute substrate content that was discharged in that period divided by the time difference between the sampling points (Equation 5). The overall specific substrate uptake rate r_s_ [gs/gx/h] was calculated as the sum of r_Glc_ and r_MSG_.

Calculation of substrate uptake rate:


(5) $$r_{s}=\frac{\left(s_{t_{n}}-s_{t_{n-1}}\right)*V_{\mathrm{Reactor}}+s_{\mathrm{in}}*V_{\mathrm{in}}-\bar{s\;}*V_{\mathrm{out}}}{t_{n}-t_{n-1}}$$

*Biomass yield*, Y_x/s_ [g_x_/g_s_], was calculated as the quotient of μ [1/h] and q_S_ [g_S_/g_x_/h].

*CO_2_ yield*, Y_CO2/s_ [Cmol CO_2_/Cmol s], was calculated as the quotient of the specific CO_2_ evolution rate [Cmol CO_2_] and q_S_ [Cmol s/g_x_/h].

*Trehalose yield*, Y_Tre/s_ [Cmol trehalose/Cmol s], was calculated as the quotient of q_Tre_ [Cmol trehalose/g_x_/h] and q_S_ [Cmol s/g_x_/h].

*Total amino acid yield*, Y_AA/s_ [Cmol amino acids/Cmol s], was calculated as the quotient of q_Tre_ [Cmol amino acids/g_x_/h] and q_S_ [Cmol s/g_x_/h].

*C-balance*, [−], was calculated as the sum of Y_x/s_, Y_CO2/s_, Y_Tre/s_ and Y_AA/s_.

### 2.7 Statistical analysis of rates and yields

All values, if not stated elsewise, are represented as means ± standard deviation. Statistical analysis of rates and yields was done using one-way variance ANOVA (*post hoc* Tukey test) in Origin Pro 2021b (OriginLab Corporation, United States). A *p*-value of ≤0.025 was denoted with a single asterisk (*).

### 2.8 RNA extraction, RNA sequencing and data analysis

For transcriptomic analysis, 2 mL samples of cell broth from the Low, High and Overfeed condition were collected in biological quintuplicates, stabilized with 3 mL RNAprotect Bacteria Reagent (Qiagen, Netherlands), and subsequently centrifuged (6,574×*g*; 4 °C, 10 min). Cell pellets were stored at -80 °C till further use. Total RNA was extracted using the RNeasy Midi Kit (Qiagen, Netherlands), including an on-column DNase treatment (Qiagen, Netherlands). Both procedures were performed according to the manufacturer’s instructions. RNA quality and quantity were analyzed using a bioanalyzer system (Agilent Technologies, United States) and the RNA 6000 Nano kit (Agilent Technologies, United States).

Ribosomal RNA depletion was performed using the PAN-Archaea riboPOOL kit (siTOOLS, Germany) using 3 μg input RNA. Sequencing libraries were constructed with the Illumina Stranded mRNA Prep Ligation Kit, using dual indices (Illumina, United States). The libraries were PCR-amplified for six cycles and purified using AMPure XP Beads (Beckman-Coulter, United States). Quality was checked with a Bioanalyzer High Sensitivity DNA Kit (Agilent Technologies, United States) and quantification was performed using qPCR according to the Illumina protocol. Finally, the libraries were pooled equimolarly, spiked with 2% PhiX and sequenced as single-read 72 on a NextSeq 500 device (Illumina, United States).

The quality and length of the sequencing reads were assessed using FastQC (Andrews, 2010). Adaptor and quality trimming were done using cutadapt (Martin, 2011) with added filtering of reads containing ambiguities or not passing the phred score threshold of 20. Quality of the remaining reads was checked using FastQC (Andrews, 2010). Trimmed sequencing reads were aligned on the *S. acidocaldarius* DSM639 genome (gca_000012285.ASM1228v1, ENSEMBL) using the STAR mapper (Dobin et al., 2013). Feature counting at the gene level was done using rsem-calculate-expression (RSEM) (Li and Dewey, 2011). All statistical analyses were performed in R using the edgeR package (Robinson et al., 2010). Feature counts were TMM-normalized, and dispersions were estimated. We used the preferred and more robust quasi-likelihood model and F-test for a more reliable error rate. Correction of the *p*-values for repeated testing (PAdj) was performed using the Benjamini-Hochberg method (Benjamini and Hochberg, 1995).

### 2.9 Metabolic modeling

Since a genome-scale metabolic model (GSMM) of *S. acidocaldarius* was not available, GSMM of *Saccharolobus solfataricus* was used instead (Wolf et al., 2016). The model was downloaded in SBML format from https://fairdomhub.org/models/225#studies. Parsimonious flux balance analysis (pFBA) simulations were done using cobrapy 0.27.0 in python 3.8.15 with cplex 22.1.0.0 as the solver. Biomass production was used as the objective function. The minimum non-growth associated maintenance energy was set to 1.9 mmol/(g*h) (Ulas et al., 2012). Exchange rates were constrained based on HPLC analysis as follows: q_Acetate_ = 0; q_Lactate_ = 0; q_Ethanol_ = 0; q_Glycerol_ = 0; q_Oxaloactetae_ = 0; q_Malate_ = 0; q_Pyruvate_ = 0; q_Citrate_ = 0. To get a representative sample of growth rates and fluxes, the lower and upper bounds of the measured exchange reactions were sampled from the interval [rate − 2*SD, rate + 2*SD]. This sampling was done 1,000 times, and pFBA was performed for each sample. The growth rates and metabolic fluxes were then averaged. Flux distributions were visualized with Escher.

## 3 Results

### 3.1 Physiology and growth of *Sulfolobus acidocaldarius* under the different growth conditions

In this manuscript, we compare three process conditions in chemostat cultivations of *S. acidocaldarius* to study the transcriptome and physiological response to high C-source concentrations. As the main focus of this study was to describe the metabolic response during the cultivation of *S. acidocaldarius* to nutrient availability, we measured and calculated process-relevant physiological variables.

**Figure 1:**
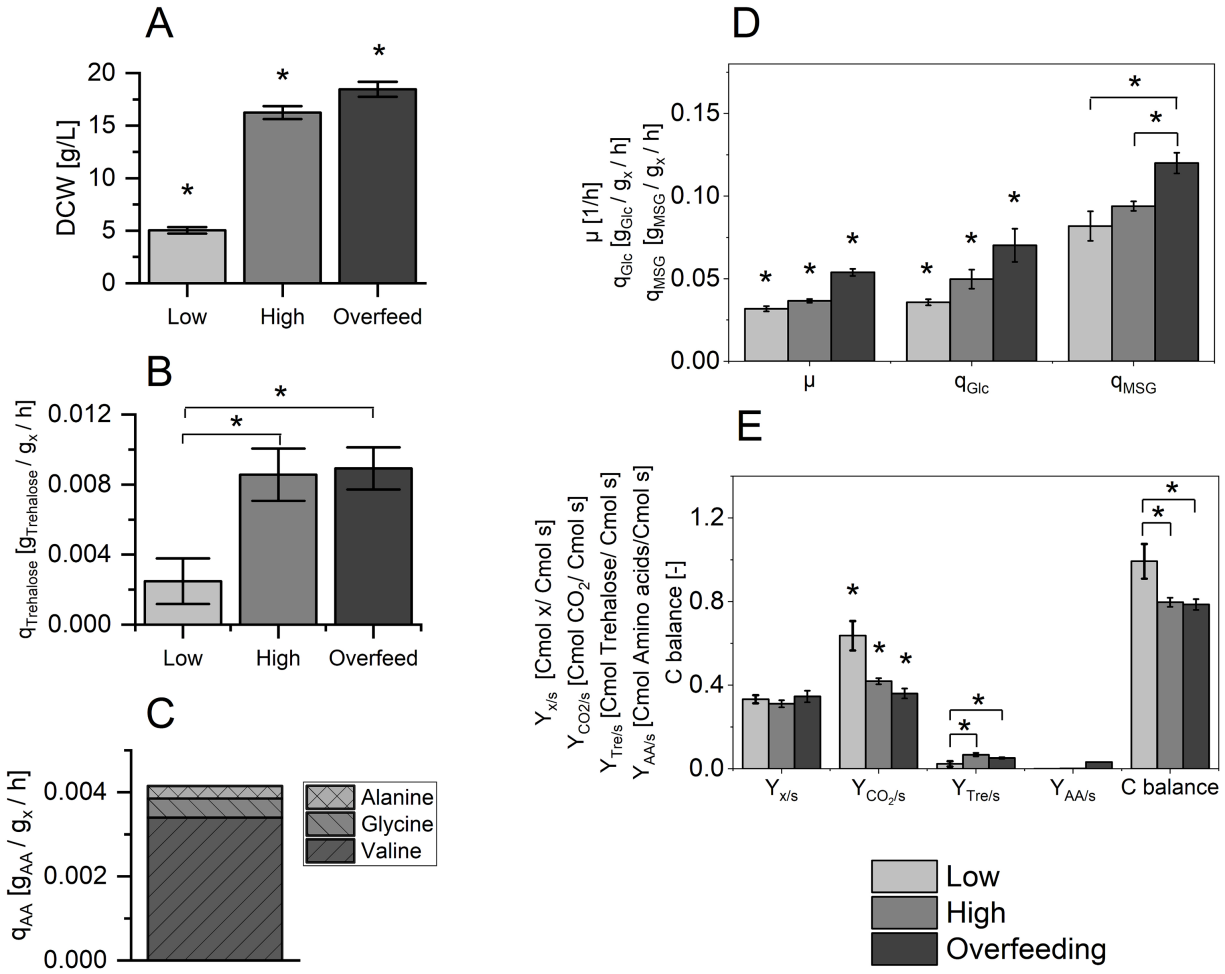
**(A)** Dry cell weight (DCW) for conditions Low, High and Overfeed. **(B)** Specific formation rate of trehalose, q_Trehalose_ [g_Trehalose_/g_x_/h] for condition Low, High and Overfeed. **(C)** Specific formation rate of amino acids, q_AA_ [g_AA_/g_x_/h] for valine, glycine and alanine for the condition Overfeed. **(D)** Specific growth rate, *μ* [1/h], specific glucose (Glc) uptake rate, q_Glc_ [g_Glc_/g_x_/h], and specific monosodium glutamate (MSG) uptake rate, q_MSG_ [g_MSG_/g_x_/h]. **(E)** Biomass yield, Y_x/s_ [Cmol x/Cmol s], CO_2_ yield, Y_CO2/s_ [Cmol CO_2_/Cmol s], trehalose yield, Y_Tre/s_ [Cmol trehalose/Cmol s], and amino acid formation yield Y_AA/s_ [Cmol amino acids/Cmol s]. Values are represented as mean ± standard deviation from five biological replicates used for transcriptomic analysis. Asterisks indicate a significant difference (*p-*value ≤0.025).

Figure 1A compares the volumetric DCW obtained at the respective sampling points. Significant differences between samples derived from all three conditions were acquired. DCW increased more than three-fold from conditions Low (DCW_Low_ = 5.0 ± 0.3 g) to High (DCW_High_ = 16.2 ± 0.6 g), while the highest DCW was obtained during the condition of Overfeed (DCW_Overfeed_ = 18.5 ± 0.6 g).

Figure 1B depicts the specific rates for the formation of trehalose (q_Trehalose_), a common secretion product of *S. acidocaldarius* (Rastädter et al., 2021). As for DCW, q_Trehalose_ increased more than three-fold when changing the process conditions from Low (q_Trehalose, Low_ = 0.003 ± 0.001 g_P_/g_s_/h) to High (q_Trehalose, High_ = 0.009 ± 0.001 g_P_/g_s_/h), but there was no difference between High and Overfeed (_qTrehalose, Overfeed_ = 0.009 ± 0.001 gP/gs/h).

As common secretion products associated to overflow metabolism could not be detected via HPLC analysis, we decided to analyze the amino acid content in the supernatant as potential secretion products at elevated nutrient availability. Figure 1C shows the secretion rates q_AA_ of three amino acids—valine, glycine and alanine—for the Overfeed. These amino acids had the highest formation rates in Overfeed, while the calculated amino acid formation rates for Low and High were comparably low. A complete overview of the formation rates for all analyzed amino acids in all three conditions is provided in Supplementary Tables S2–S4. Valine was the amino acid secreted at the highest rate, followed by glycine and alanine.

Figure 1D compares the specific growth rate *μ* and the specific substrate uptake rates for glucose (q_Glc_) and MSG (q_MSG_) for the three process conditions. With increasing nutrient availability, μ increased from 0.031 ± 0.002 h^−1^ (Low) to 0.037 ± 0.001 h^−1^ (High) to 0.054 ± 0.002 h^−1^ (Overfeed). These values represent calculated specific growth rates according to Equation 1, which must be considered when evaluating μ_Overfeed_. In a chemostat in steady state, μ is equal to the applied dilution rate. Due to the short duration of the Overfeed condition and the consequences on the process performance, it represents a substrate pulse in a stricter sense and not a steady state. Comparing the specific substrate uptake rates, q_MSG_ exceeded q_Glc_ for all three cultivation conditions. With increasing nutrient availability, the uptake rate for both C-sources increased. Interestingly, this increase was not proportional, as their ratio q_MSG_/q_Glc_ decreased from 2.3 (Low) over 1.9 (High) to 1.7 (Overfeed).

Figure 1E shows the biomass yield (Y_x/s_), the CO_2_ yield (Y_CO2/s_), the trehalose yield (Y_Tre/s_), the total amino acid formation yield (Y_AA/s_), and the C-balance. While Y_x/s_ was similar for all conditions, Y_CO2/s_ dropped significantly for High and Overfeed. In Low, Y_CO2/s_ accounts for more than 60% of the total carbon in the C-balance. Compared to Y_CO2/s_, the higher product formation yields for trehalose and amino acids only contributed minorly to the total C-balance, which was reflected in the not closing C-balance for High (0.80 ± 0.02) and Overfeed (0.79 ± 0.02). Therefore, the presence of undetected metabolites and/or a changing biomass composition were likely for High and Overfeed.

Table 1 shows the accumulation rates of the two C-sources contained in the VD Medium. MSG accumulation in the broth was more than 35 times greater than for glucose.

**Table 1:** Accumulation rate of the two C-sources monosodium glutamate (MSG) and glucose during Overfeeding.

C-source	Accumulation rate [g/L/h]
MSG	0.18 ± 0.07
Glucose	0.0048 ± 0.0005

### 3.2 Transcriptomic analysis

#### 3.2.1 Overall results of RNA-seq analysis and arCOG categories

To differentiate between genes that were differentially transcribed due to potential stress conditions associated with the cultivation at elevated cell densities and those activated due to the response to high nutrient concentrations, we primarily compared genes that were either significantly up- or downregulated in both Low and High compared to Overfeed. We focused our analysis on changes in the central carbon metabolism originating from the two C-sources during cultivation, MSG and glucose. Additionally, as the C-balance did not close for High and Overfeed (Figure 1E), we analyzed genes related to the accumulation of intracellular carbon, e.g., to function as an energy reservoir, such as glycogen and fatty acids. The complete results of the RNA-seq analysis is provided in Supplementary material 2. For each comparison, the number of statistically significant differentially expressed genes having an adjusted *p*-value <0.05 and at least a doubled (upregulated, log_2_FC ≥ 1) or halved (downregulated, log_2_FC ≤ −1) expression level are reported in Table 2. Observing the Overfeed condition, a substantial portion of gene upregulation can be recognized. The expression of 215 and 81 genes was enhanced compared to conditions Low and High, respectively.

**Table 2:** Number of statistically significant differentially expressed genes per comparison.

Designator	Total	Upregulated	Downregulated
Overfeed/Low	255	215	40
Overfeed/High	93	81	12
Low/High	69	15	54

The following thresholds were applied: corrected *p*-value (PAdj) < 0.05 and either log_2_FC > =1 for upregulated genes or log_2_FC = <−1 for downregulated genes.

**Figure 2:**
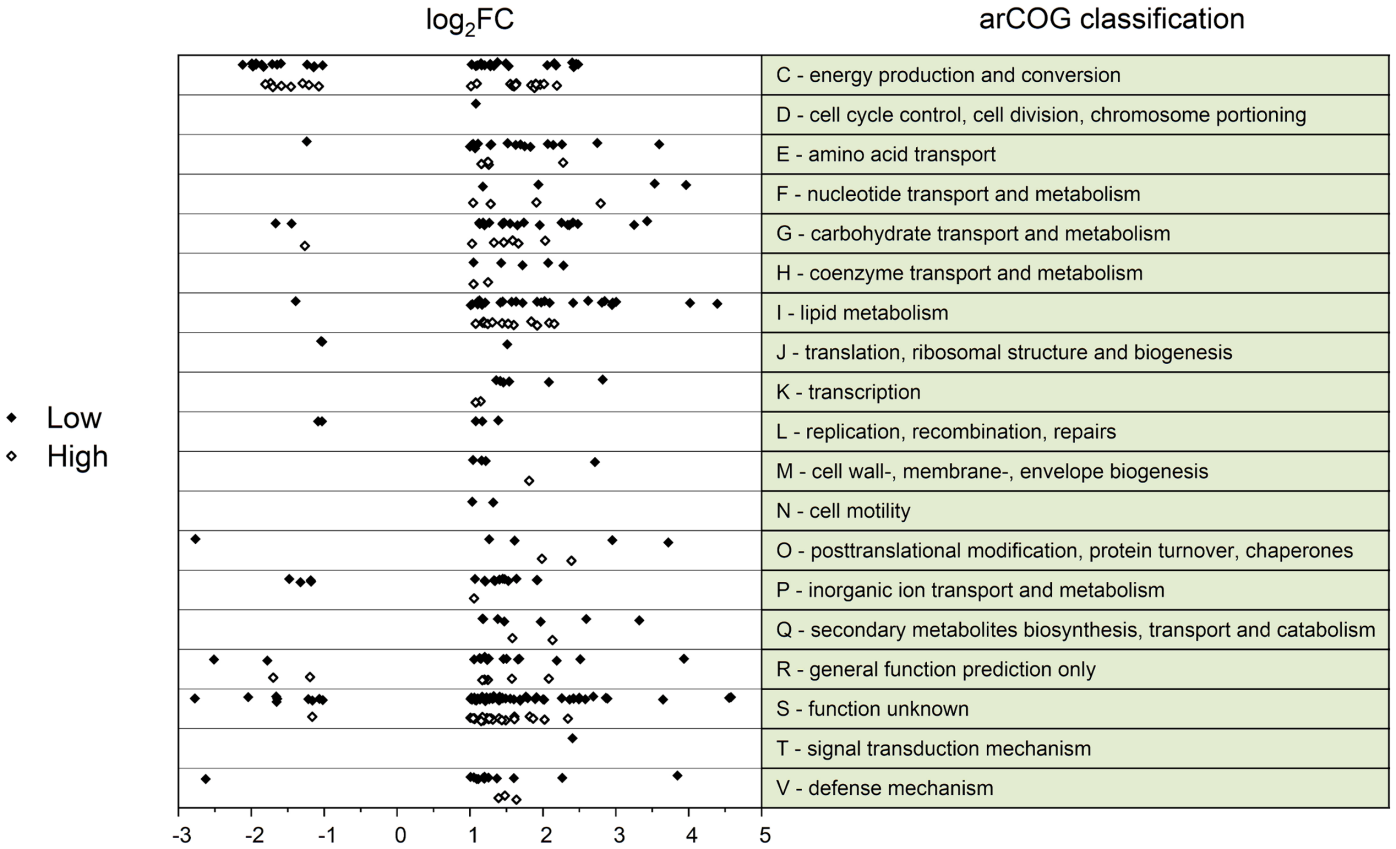
ArCOG classification of differentially transcribed genes (log_2_ fold change (log_2_FC) > 1; log_2_FC < −1) for the conditions Low (black diamond) and High (white diamond), compared to the condition Overfeed. Positive log_2_FC values indicate higher transcription in Overfeed, negative log_2_FC values indicate lower transcription in Overfeed.

The differentially transcribed genes were categorized according to their archaeal cluster of orthologous genes (arCOG) (Figure 2). Only two arCOG classes, namely class C (energy production and conversion) and S (unknown function), were associated with both substantial up-and down-regulation of more than three genes. Additionally, significant changes in lipid metabolism (arCOG class I) were recognized in Overfeed compared to Low and High. This involved both genes active in the metabolism of fatty acids and 3HP-4HB cycle-associated genes associated with the metabolism of small-chain acyl groups (Table 3). Genes involved in amino acid transport (arCOG class E) and carbohydrate transport (arCOG class G) were also upregulated in Overfeed (Figure 2; Table 3).

**Table 3:** Changes in transcript levels of *Sulfolobus acidocaldarius* in response to overfeeding conditions identified by RNA sequencing with log_2_ fold change (log_2_FC) > 1 and log_2_FC < −1.

arCOG	Locus	(Predicted) function	log_2_FC compared to Low	log_2_FC compared to High
C—Energy production	*saci_2258*	SoxEFGHIM complex	2.1	1.6
	*saci_2259*		2.2	1.6
	*saci_2260*		2.4	1.8
	*saci_2261*		2.5	1.9
	*saci_2262*		2.5	1.9
	*saci_2263*		2.2	1.6
C—Energy production	*saci_2085*	SoxABCDL complex	−2.0	NA
	*saci_2086*		−1.9	NA
	*saci_2087*		−2.0	NA
	*saci_2088*		−2.0	NA
	*saci_2089*		−2.0	NA
	*saci_2090*		−1.7	NA
C—Energy production	*saci_1858*	CbsAB-SoxLN-OdsN	−1.9	NA
	*saci_1859*		−1.6	NA
	*saci_1860*		−2.1	NA
	*saci_1861*		−1.9	NA
	*saci_1862*		−1.8	NA
E—Amino acid transport	*saci_1127*	Na+/proline symporter	2.3	1.2
	*saci_1745*	Amino acid transporter	2.7	NA
	*saci_1760*	ABC-type transport system	2.1	1.2
	*saci_1835*	Amino acid transporter	2.4	1.5
	*saci_2181*	Amino acid transporter	2.1	NA
G—Carbohydrate transport and metabolism	*saci_0675*	Conserved membrane protein	2.5	1.7
	*saci_1003*	Conserved membrane protein	−1.7	−1.3
	*saci_1058*	Sugar kinase	2.4	1.3
	*saci_1162*	Membrane-bound alpha-amylase	2.3	1.6
	*saci_1163*	Sugar transport system permease	1.3	NA
	*saci_1164*	Conserved transport protein	1.5	NA
	*saci_1165*	Sugar-binding periplasmic protein	1.7	NA
	*saci_1166*	ABC transporter ATPase	1.4	NA
	*saci_1167*	Conserved CBS domain protein	1.7	1.2
	*saci_1760*	Conserved membrane protein	2.1	1.2
	*saci_2122*	Hypothetical protein	2.0	1.0
I—lipid metabolism	*saci_1054*	Acyl-CoA synthetase	3.0	1.6
	*saci_1134*	3-Hydroxyacyl-CoA dehydrogenase	2.6	1.2
	*saci_2148*	Acyl-CoA synthetase	2.1	1.1
	*saci_2208*	3-Hydroxyacyl-CoA dehydrogenase	2.8	1.4
	*saci_2209*	Acetyl-CoA acetyltransferase	4.0	2.1
	*saci_2211*	Acyl-CoA synthetase	2.4	2.1
	*saci_2219*	Sterol carrier protein	2.0	1.5
	*saci_2232*	Acetyl-CoA acetyltransferase	2.8	1.8
	*saci_2233*	Acetyl-CoA acetyltransferase	3.0	1.9
	*saci_2234*	Acyl-CoA dehydrogenase	2.9	1.3
	*saci_2235*	Acyl-CoA synthetase	4.4	2.2
Others	*saci_2188*	Ribonucleoside-diphosphate reductase	3.5	1.9
	*saci_2212*	Ribonucleotide reductase, small chain	4.0	2.8
	*saci_2293*	2-Keto-4-pentenoate hydratase/2-oxohepta-3-ene-1,7-dioic acid hydratase	2.6	1.6
	*saci_2294*	Aromatic ring hydroxylase	3.3	2.1
	*saci_2295*	Catechol 2,3-dioxygenase	3.6	2.3

NA, not applicable.

#### 3.2.2 Incorporation of glucose and glutamate into the carbon metabolism

The gene cluster *saci_1162–1166*, annotated as the putative glucose ABC transporter (Joshua et al., 2011), was upregulated in Overfeed. Additionally, we found several genes encoding membrane-bound transporters of carbohydrates that were transcribed differentially with excessive C-source availability. *saci_0675*, *saci_1003*, *saci_1058*, *saci_1760*, and *saci_2122* (Buetti-Dinh et al., 2016; Chauhan et al., 2021) showed substantial upregulation in the Overfeed condition. For the further metabolization of glucose, *S. acidocaldarius* utilizes the branched Entner Doudoroff (ED) pathway to generate pyruvate, eventually entering the tricarboxylic acid (TCA) cycle (Quehenberger et al., 2017). No regulation for genes involved in the ED pathway could be observed. XylR, annotated as an activator of the pentose/arabinose inducible regulon (van der Kolk et al., 2020), was upregulated in the Overfeeding condition. No precise description of transporter systems involved in glutamate transport is yet known. Vetter described several putative transporters involved in glutamate transportation determined in a transcriptional study comparing the growth on amino acids and glucose (Vetter, 2020). Among these, the differential expression of *saci_1127* with increasing nutrient availability and of *saci_1835* when comparing Overfeed against High and Low was observed. Glutamate enters the TCA cycle via oxidative deamination by glutamate dehydrogenase (*saci_0155*) or via transamination catalyzed by a transferase. No differential expression was observed for glutamate dehydrogenase, while one aminotransferase class III (*saci_2137*) was slightly upregulated for the Overfeed compared to Low. Transcription levels of *saci_0368* and *saci_0369* encoding 5-oxoprolinase (Vetter et al., 2019), involved in the degradation of pyroglutamate, a conversion product of glutamate at high temperatures and low pH, were not regulated differentially.

#### 3.2.3 Energy production and conversion

Although early reports suggest a possible chemolithoautotrophic lifestyle, *S. acidocaldarius* only utilizes organic compounds for energy production (Zeldes et al., 2019). This primarily involves the tricarboxylic acid cycle and respiration. On a transcript level, no significant changes could be observed for the enzymes involved in the TCA cycle. The respiratory chain of *S. acidocaldarius* compromises NADH dehydrogenase, succinate dehydrogenase, quinones, cytochromes bc1 complex SoxNL-CbsAB-OdsN (*saci_1858–1862*), and terminal oxidases. Electrons from NADH and succinate dehydrogenase are transferred through this chain, ultimately generating a pH gradient for ATP synthesis. Three terminal oxidases are known: SoxABCDL (*saci_2086–2090*) and DoxBCE (*saci_0097–0099*), possessing the same redox potential, as well as SoxEFGHIM (*saci_2258–2263*) with the ability to transfer two protons per electron (Bischof et al., 2019). The cytochrome bc1 SoxNL-CbsAB-OdsN was downregulated, while SoxEFGHIM was upregulated in the Overfeed condition compared to Low and High (Table 3). SoxABCDL was downregulated in Low compared to High and Overfeed.

#### 3.2.4 Lipid metabolism

Besides the secretion of metabolites, the non-closing C-balance for High and Overfeed indicate the storage of excess carbon. While isoprenoid-based ether lipids are incorporated in the archaeal cell membrane, the occurrence of fatty acids has been demonstrated and can take over a crucial role in the functionality of archaeal enzymes (Corcelli et al., 1996). Schmerling et al. elucidated the genes involved in fatty acid synthesis in *S. acidocaldarius* via an ACP-independent reaction involving a fatty acid synthase complex (*saci_1085*, *saci_1104*, *saci_1115*, *saci_1120/1121*) (Schmerling et al., 2024). Genes encoding the proposed fatty acid synthase were not differentially transcribed (Schmerling et al., 2024); however, other genes with putative involvement in fatty acid metabolism were. Another gene cluster, potentially associated with fatty acid synthesis, is located at *saci_1103*-*saci_1126* (Dibrova et al., 2014; Wang et al., 2019). Upregulation of several genes was observed. Furthermore, the upregulation of several acetyl-CoA acetyltransferases may indicate a reorganization of the lipid composition of the membrane, as acetoacetate is an intermediate for mevalonate biosynthesis, a precursor for isoprenoid biosynthesis (Matsumi et al., 2011).

#### 3.2.5 Glycogen metabolism

Besides lipids, polysaccharides are the most common form of carbon and energy preservation in biology. Glycogen is produced intracellularly by *S. acidocaldarius* by genes belonging to the *glg* operon (Lee et al., 2021). However, no differential transcription of genes of this cluster (*saci_1197*-*saci_1201*) was recognized.

#### 3.2.6 Other highly transcribed genes

The genes that experienced one of the highest upregulations in Overfeed were two genes coding for the β subunit of ribonucleotide reductase (*saci_2188* and *saci_2212*). Log_2_FC of Low (3.5 and 3.9) and High (1.9 and 2.8) compared to Overfeed indicate a substantial upregulation, while no differential gene expression was recognized for the α subunit. High transcription of proteases thermopsin (*saci_1714*) and subtilase (*saci_1147*) was found. Furthermore, a high upregulation of transcripts encoding enzymes involved in the catechol pathway could be recognized (*saci_2293–2295*).

### 3.3 Flux analysis

The absence of significant transcript expression changes in the central carbon metabolism (e.g., ED-pathway and TCA cycle), as revealed by RNA-seq analysis, prompted the implementation of a parsimonious Flux Balance Analysis (pFBA). This approach aimed to uncover the metabolic flux distributions governing these pathways under the observed conditions. Due to the absence of a metabolic model for *S. acidocaldarius*, a model of *Sa. solfataricus* P2 was tested for applicability (Wolf et al., 2016). Together with *S. acidocaldarius*, it can potentially be considered the most studied crenarchaeote, with a genome size of 2,992,245 bp (*Sa. solfataricus* P2) compared to 2,225,959 bp (*S. acidocaldarius*). The organisms share approximately 90% of metabolic reactions (BioCyc database). Predictions by the model for the specific growth rates showed that the simulation could successfully reproduce the experimentally determined growth rates and are provided in Supplementary Figure S1.

**Figure 3:**
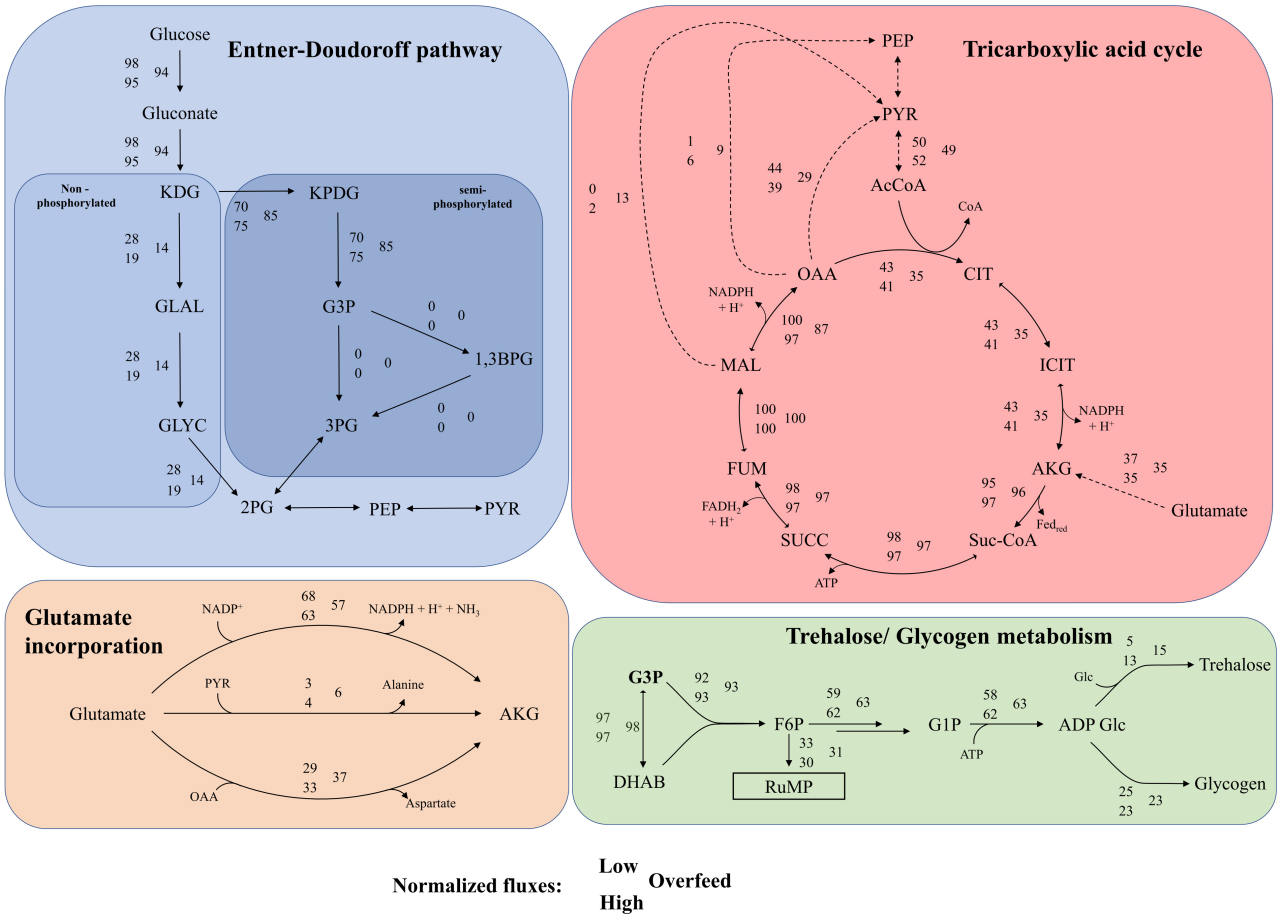
Results of parsimonious flux balance analysis for Entner Doudoroff (ED) pathway, tricarboxylic acid cycle, glutamate, glutamate incorporation and trehalose/glycogen metabolism for the three conditions: Low, High and Overfeed. Values are normalized, details see further in the caption. Top left in blue: Non-phosphorylated and semi-phosphorylated ED pathway. Fluxes of individual reactions are normalized on the flux of glucose uptake. Normalized fluxes for the reaction 3PG <-> 2PG, 2PG <-> PEP and PEP <-> PYR could not be provided as they are predicted to run in different directions for the three conditions. Top right in red: Tricarboxylic acid (TCA) cycle and selected TCA cycle depleting reactions. Fluxes are normalized on the maximal flux in the TCA cycle; Bottom left in orange: Mechanisms of anaplerotic reactions of glutamate to alpha-ketoglutarate. Fluxes are normalized on total flux for glutamate incorporated into the TCA cycle; Bottom right in green: mechanism of glycogen/trehalose formation outgoing from glycerol-3-phosphate (G3P). Fluxes are normalized on flux for G3P formation. KDG, 2-keto-3-deoxygluconate; KPDG, 2-keto-3-deoxy-6-phosphogluconate; G3P, glycerol-3-phosphate; 1,3BG, 1,3-bisphosphoglycerate; 3PG, 3-phosphoglycerate; GLAL, glyceraldehyde; GLYC, glycerol; 2PG, 2-phosphoglycerate; PEP, phosphoenolpyruvate; PYR, pyruvate; AcCoA, acetyl-coenzyme A; CIT, citrate; ICIT, isocitrate; AKG, alpha-ketoglutarate; Suc-CoA, succinyl coenzyme A; SUCC, succinate; FUM, fumarate; MAL, malate; OAA, oxaloacetate; DHAB, dihydroxyacetone phosphate; F6P, fructose 6-phosphate; G1P, glucose 1-phosphate; ADP-Glc, ADP-glucose.

The pFBA of the three conditions revealed several metabolic patterns, as summarized in Figure 3. When using the two C-source-containing VD Medium, the metabolic roles of MSG and glucose appear to be distinct. For Low, minor flux and for High and Overfeed, no flux was observed in the reactions of the semi-phosphorylated ED pathway converting glycerol-3-phosphate (G3P) to 1,3-bisphosphoglycerate (1,3BP)/3-phosphoglycerate (3PG) and phosphoenolpyruvate (PEP) to pyruvate. For High and Overfeed, a flux originating from PEP toward 2-phosphoglycerate (2PG) was predicted, suggesting an overall flux from intermediates of the TCA cycle. For all three conditions, the non-phosphorylated pathway was predicted to be the disfavored pathway for glucose utilization, whereas pFBA suggested a substantially decreased contribution for the Overfeed condition. Detailed metabolic maps for Low, High, and Overfeed samples are provided in Supplementary Figures S2–S4.

Consequently, glucose was primarily used for the biosynthesis of intermediates and trehalose/glycogen production rather than as an energy source. Here, G3P appears to take over a vital role as the precursor for trehalose/glycogen metabolism and the reversed ribulose monophosphate (RuMP) pathway. Only for Low, the ED pathway ran entirely, resulting in a net gain of ATP, whereas, for High and Overfeed, ATP was consumed. The semi-phosphorylated ED pathway gained importance with increasing nutrient availability, minimizing NADP^+^ consumption. In contrast, MSG was predicted to be the primary energy source, entering the TCA cycle as alpha-ketoglutarate mainly via oxidative deamination, with transamination playing a secondary role. In Overfeed samples, substantial transamination was evident, depleting oxaloacetate (OAA). The TCA cycle was balanced in Low and High, with OAA being converted to pyruvate across all conditions.

Additionally, OAA was converted to PEP in High and Overfeed. Malate was predominantly converted to pyruvate in Overfeed, with minimal conversion in High. For Overfeed samples, PEP was converted to 2-phosphoglycerate (2PG), a pathway only slightly active in High. Pyruvate was significantly depleted in Overfeed due to valine formation, while this process was minimal in High. Overall, the High condition was on the brink of overflow metabolism, showing intermediate metabolic activities between balanced metabolism (Low and High) and overflow metabolism (Overfeed). Substantial amounts of acetyl-CoA derived from pyruvate were found for Overfeed, with almost 30% not entering the TCA cycle. Interestingly, the saturation of the TCA cycle appeared to be so high that a glycogenetic role of PEP was predicted (see Supplementary material 2).

## 4 Discussion

This study assessed the physiological, transcriptome, and metabolic response of *S. acidocaldarius* to different nutrient availability. While the behavior of mammalian, fungal, and bacterial cells in nutrient-rich environments has been extensively studied for several decades, the effects on archaeal organisms, especially Crenarchaeota, have remained largely unexplored. This highlights the significant challenges and limitations in archaeal biotechnology. Despite recent advances in genetic manipulation techniques for archaea, there are still considerable obstacles compared to those for their eukaryotic and bacterial counterparts. These difficulties are partly due to the extreme conditions required and the relatively low growth rates associated with many archaeal species. This underscores the importance of improving cultivation methods for archaea and emphasizes the relevance of our efforts in this field (Quehenberger et al., 2019; Rastädter et al., 2021; Sedlmayr et al., 2024a).

Therefore, we decided to perform chemostat cultivations, allowing the study of cells under strictly defined physiological steady states with theoretical unchanging concentrations of intra-and extracellular molecules (Adamberg et al., 2015). Consequently, the samples derived from equal process parameters are associated with a high degree of homogeneity, allowing the almost unaltered study of the temporal dynamics of cells when compared to samples derived from simple batch experiments (Nieto-Taype et al., 2020). The bioprocessing of extremophilic archaea has experienced significant progress in recent years, mainly due to their enormous potential for the biopharmaceutical industry (Quehenberger et al., 2017). This development is exemplified by the development of a defined medium for the cultivation of several *Sulfolobus* strains (Quehenberger et al., 2019) and the determination of critical process parameters for the continuous cultivation of *S. acidocaldarius* (Rastädter et al., 2021).

Building on these achievements, we analyzed three states during chemostat cultivations—Low, High, and Overfeed—each characterized by adjustments in total flow rate and nutrient concentration in the feed, resulting in different C-source availability in the bioreactor. “Low” represents the baseline condition with minimal stress on the organism, allowing for the examination of the metabolic state without overflow metabolism (40% of μ_max_). In “High,” stress was introduced to the system by increasing both TFR and nutrient concentration, enhancing the specific growth rate to almost 50% of μ_max_. This allowed for the examination of the cellular response toward higher nutrient availability without causing C-source accumulation and provided a middle ground to understand the transition from normal to stressed metabolic states. “Overflow” represents a high stress condition, where maximization of the nutrient availability leads to C-source accumulation and significant overflow metabolism. Here, cultivations were conducted at approximately 70% of μ_max_.

By adjusting the respective process parameters, cell density was enhanced from 5.0 g/L (Low) up to 18.5 g/L (Overfeed). The significantly increased μ also reflects the increased biomass concentration. Consequently, with enhanced nutrient availability, the specific substrate uptake rates for glucose and MSG increased. Noteworthy, the ratio between q_MSG_ and q_Glc_ was decreasing from Low to Overfeed. With increasing nutrient availability, the preference for the two C-sources changed, eventually leading to an almost 40-fold higher accumulation rate for MSG than for glucose. This observation is striking, as the Monod substrate affinity constant of *S. acidocaldarius* toward MSG has been described to be 7-fold higher than for glucose (Rastädter et al., 2021).

Two transporters, putatively involved in glutamate transport, were upregulated, whereas *saci_1835* was differentially transcribed only for the Overfeed condition. Reasons for the enhanced MSG accumulation might be a saturation of transporters for MSG or a changing regulatory mechanism. While we cannot conclude the degree of saturation, the reason behind the accumulation of MSG is likely a saturated TCA cycle and/or the accumulation of harmful metabolites. While glucose utilization exploits flexibility due to the branching of the ED pathway, the incorporation of MSG into the metabolism is directly linked to its transformation to AKG and utilization via the TCA cycle, resulting in the production of ATP. ATP has already been identified as harmful at elevated concentrations (Li et al., 2022). As predicted by pFBA, the cells performed substantial efforts in the depletion of intermediates of the TCA cycle with increasing nutrient availability, with higher fluxes and more active reactions to deplete intermediates of the TCA cycle. The putative glucose ABC transporter was upregulated in Overfeed, potentially explaining the efficient glucose utilization and its low accumulation rate. *S. acidocaldarius* utilizes a modified ED pathway for glucose utilization, including two branches. In *Sa. solfataricus*, the semi-phosphorylative pathway is used for glycogenetic functions, while the glycolytic branch is completed in the non-phosphorylative pathway (Kouril et al., 2013). Thereby, the formation of thermolabile intermediates of the semi-phosphorylative ED pathway, such as GAP, DHAB and 1,3 BPG, is avoided (Schmerling et al., 2022). According to pFBA, the flux toward the non-phosphorylative ED pathway was decreased in Overfeed, increasing glycogenetic utilization of glucose. Thereby, the incorporation of additional carbon into the TCA cycle and additional ATP production was prevented. It can only be speculated what the consequence higher fluxes of semi-phosphorylative ED pathway have on intracellular methylglyoxal concentration, a non-enzymatical decomposition product of GAP and DHAB. Methylglyoxal is known to cause dicarbonyl stress (Schmerling et al., 2022).

The constant biomass yield observed across the three conditions suggested that nutrient availability and potential overflow metabolism did not impact biomass production, indicating a robust metabolic regulation. Previous studies showed that process parameters, such as pH and temperature, are critical determinants of biomass yield in *S. acidocaldarius* (Cobban et al., 2020; Rastädter et al., 2021). The constant Y_x/s_ implies that the biomass concentration of *S. acidocaldarius* could potentially be boosted if a different feeding strategy was applied, such as exponentially increasing the substrate concentration. Additionally, since the MSG concentration in the VD Medium was close to the maximal metabolic capacity, increasing the glucose concentration in the feed might help sustain high carbon influx. In contrast to the biomass yield, CO_2_ yield dropped severely with increasing nutrient availability from 0.64 Cmol/Cmol (Low) to 0.36 Cmol/Cmol (Overfeed). Such observations strongly indicate an active overflow metabolism, where an increased anabolic activity favors the production of various excreted metabolites, reducing the amount of carbon funneled through oxidative pathways (Valgepea et al., 2010). Accordingly, trehalose production, a common secretion product associated with the cultivation of *Sulfolobales* (Martins et al., 1997), was enhanced. Hence, flux for the glycogenetic ED pathway was increased. However, no difference between High and Overfeed could be obtained for q_Trehalose_. A lagging formation and secretion of trehalose might be the reason for similar trehalose production rates (Stracke et al., 2020). In addition to trehalose, amino acids—particularly valine, glycine, and alanine—were found as secretion products in the cell broth. The secretion of amino acids may serve multiple purposes: Firstly, as products of pyruvate and other metabolic intermediates, they help reduce intracellular concentrations of these metabolites, alleviating flux toward the already saturated TCA cycle. Secondly, their synthesis and secretion help to manage increased NADPH levels derived from the ED pathway due to the high glucose flux, thereby maintaining redox balance within the cell. Thirdly, the secretion of these amino acids helps regulate intracellular nitrogen balance by removing excess nitrogen in the form of amino groups, thus maintaining nitrogen homeostasis. The non-closing C-balance indicates the presence of other compounds that are produced. As the HPLC method can detect several metabolites, we checked our chromatograms and compared the retention times of common secretion products. Therefore, we could exclude the secretion of metabolites, such as lactate, acetate, ethanol, glycerol, oxaloacetate, malate, pyruvate and citrate.

Apart from the secretion of metabolites, intracellular carbon storage may help explain the non-closing C-balance. Glycogen has been reported to serve as a central carbon storage in several *Sulfolobales* species (König et al., 1982). No differential expression of the *glg* operon could be detected by RNA-seq (Lee et al., 2021). However, pFBA predicts substantial flux for the glycogen pathway for High and Overfeed. Apart from polysaccharides, lipids are commonly used energy reservoirs in nature. Although the incorporation of ether lipids into the membrane is one distinct feature of the archaeal domain, the abundance of fatty acid components has long been known (Langworthy et al., 1974). Their exact role in archaea is still a matter of debate, while a vital role in the metabolism is likely, as the association of fatty acids to specific proteins is crucial for functionality (Corcelli et al., 1996). Recently, the pathways for fatty acid synthesis and β-oxidation in *S. acidocaldarius* have been described (Schmerling et al., 2024). As cultivation of *S. acidocaldarius* on short-chain fatty acids (C_4_ and C_6_) has been demonstrated experimentally (Wang et al., 2019), the utilization of fatty acids as an energy reservoir is likely. Therefore, fatty acid anabolism could be a plausible contributor to the missing carbon. Genes associated with the reconstructed fatty acid synthesis have not been differentially transcribed (Schmerling et al., 2024), however other genes with putative involvement in the fatty acid metabolism (*saci_1103*-*saci_1126*) (Dibrova et al., 2014; Wang et al., 2019). Besides, genes from the cluster *saci_2231–2235* were upregulated, encoding acyl-CoA dehydrogenases, acetyl-CoA acetyltransferase and acyl-CoA synthetase. This cluster was also associated with downregulation during cultivations on xylose compared to a mixture of xylose and NZ-amines (Wagner et al., 2018). One can speculate on the role of excess amino acids in the formation of fatty acids in archaea. Additionally, the biosynthesis of fatty acids is accompanied by the depletion of acetyl-CoA, which alleviates the oversaturation of the TCA cycle by depleting the intermediate and hinders its accumulation. Furthermore, due to the consumption of NADPH during fatty acid synthesis, fatty acid synthesis can serve as a measurement to counteract redox imbalance.

The increased transcription values for acetyl-CoA acetyltransferases might indicate a regulation of ether lipids in the membrane. Acetoacetyl-CoA is a precursor for mevalonate synthesis, with mevalonate ultimately being the starting point for the biosynthesis of isoprenoids (Matsumi et al., 2011). The membrane composition in *S. acidocaldarius* is heavily dependent on the applied growth rate (Rastädter et al., 2020). Studies show decreased cyclopentane rings in the tetraether lipid and the share of diether lipids with increasing nutrient availability (Bischof et al., 2019; Quehenberger et al., 2020). Consequently, in a nutrient-rich environment, membrane permeability increases due to decreased rings and decreased share of diether lipids.

Proton transporters from the respiratory chain are involved in the maintenance of pH homeostasis (Baker-Austin and Dopson, 2007). As found for Overfeed, the overexpression of SoxEFGHIM terminal oxidase was accompanied by the translocation of two protons compared to only one proton translocated using the other two terminal oxidases, SoxABCDL and DoxBCE. Interestingly, this was also observed during nutrient depletion (Bischof et al., 2019), indicating increased intracellular acidification. The overexpression of SoxEFGHIM was accompanied by the overexpression of several permeases and symporters, such as *saci_0383*, *saci_1745* (both Na^+^/proline symporter) and *saci_2039* (purine-cytosine permease) in the Overfeed condition. These transporters might be involved in cytoplasmic buffering as a response to the increased acidification (Bischof et al., 2019; Helmecke, 2019). The simultaneous downregulation of cytochrome SoxNL-CbsAB-OdsN in Overfeed indicated a reduced overall respiration rate, likely due to lower energy demand due to the oversaturated TCA cycle.

Among the most upregulated genes was the cluster encoding proteins annotated to the catechol pathway (*saci_2293*-*saci_2295*). Similarly, Benninghoff and coworkers noticed a high upregulation of this cluster in *S. acidocaldarius* upon exposure to organic solvents (Benninghoff et al., 2021). While these genes are involved in the degradation of aromatic amino acids, in the context of this study, it is possible that they were also involved in the degradation of aromatic compounds in general, helping in the management of reactive oxygen species.

Additionally, according to RNA-seq, *saci_2188* and *saci_2212* were the top upregulated genes, with a fold change of 3.5/1.9 and 4.0/2.8 (Low/High) compared to Overfeed. As ribonucleotide reductases are crucial in DNA synthesis, this might indicate an increased demand for cellular repair machinery.

## 5 Conclusion

In this study, we gathered valuable insights into the basic metabolic and physiological responses of *S. acidocaldarius* upon excess nutrient concentrations. Despite a lower Monod affinity constant for MSG, its accumulation surpassed that of glucose. While glucose primarily undergoes glycogenesis, MSG integrates directly into the TCA cycle, leading to saturation and enhanced pyruvate levels. At the same time, metabolic regulation appeared to be robust, indicated by consistent biomass yields across the different conditions. Therefore, an adaption of glucose and MSG concentration in the VD Medium during high cell density cultivations might help to boost biomass concentrations of *S. acidocaldarius*. The non-closing C-balance speaks for the presence of other metabolites, with HPLC analysis excluding common secretion products. For the first time, we described the secretion of amino acids by *S. acidocaldarius*, with valine being secreted at rates similar to the common glycogenesis product trehalose. Furthermore, a strong impact on lipid metabolism was observed, suggesting intracellular carbon and energy storage and change in membrane composition. Also, changes in respiratory protein transcription suggested intracellular acidification at high nutrient concentrations, with identified transporters aiding cytoplasmic buffering. Overall, the study highlighted the metabolic flexibility and robust regulatory mechanisms in *S. acidocaldarius*. While in yeasts, bacteria and mammalian cells, excessive nutrient concentrations trigger different metabolic pathways, it appears that *S. acidocaldarius* tries to maintain its normal metabolism by reducing TCA intermediates, storing energy via fatty acid synthesis, secreting amino acids, and maintaining high glycogenesis. The absence of differentially expressed genes in key metabolic pathways prompts questions about the regulation at other levels, such as post-transcriptional, and suggests avenues for future research in understanding these regulatory mechanisms.

## Data Availability

The code, metabolic model, and input data were deposited to GitHub at https://github.com/diana-sz/SaciOverflow. Raw RNA-seq data have been deposited in the European Nucleotide Archive at EMBL-EBI under accession number PRJEB79913.
