# Supplementary material for: Impact of nutrient excess on physiology and metabolism of Sulfolobus acidocaldarius
Source: Front Microbiol. 2024 Oct 4;15:1475385. doi: 10.3389/fmicb.2024.1475385 (PMC11486757; doi:10.3389/fmicb.2024.1475385)
Supplement: Supplementary file 1 [file Data_Sheet_1.pdf]

Supplementary Material

Supplementary Table S1: Limit of quantification of the amino acids

	**Limit of quantification [µmol/L]**
Glycine	10.4
Alanine	1.04
Proline	5.21
Valine	1.04
Threonine	0.104
Isoleucine	0.104
Aspartate	0.104
Lysine	0.104
Phenylalanine	0.104
Leucine	0.104
Methionine	1.04
Histidine	0.104
Arginine	1.04
Tyrosine	0.104
Cysteine	1.04

Supplementary Table S2: Amino acid formation rate r_p, specific amino acid formation rate q_p and the amino acid yields determined via LC-ESI-MS analysis of the supernatant of condition "Low". Measurement was performed for 15 amino acids; n.d. indicates that the concentration for the amino acid was below the limit of quantification of the analytical method. The notation n.d. stands for "not determined" due to an amino acid concentration below the limit of quantification (see Supplementary Table S1).

	**r_p [gp/h]**	**q_p [g_p/g_x/h]**	**Yp/s [g_p/g_s]**	**$Y_{p/s}$ [Cmol p/Cmol s]**
Glycine	n.d.	n.d.	n.d.	n.d.
Alanine	n.d.	n.d.	n.d.	n.d.
Proline	n.d.	n.d.	n.d.	n.d.
Valine	n.d.	n.d.	n.d.	n.d.
Threonine	2.07E-06	2.61E-07	3.38E-06	3.70E-06
Isoleucine	n.d.	n.d.	n.d.	n.d.
Aspartate	n.d.	n.d.	n.d.	n.d.
Lysine	n.d.	n.d.	n.d.	n.d.
Phenylalanine	n.d.	n.d.	n.d.	n.d.
Leucine	n.d.	n.d.	n.d.	n.d.
Methionine	n.d.	n.d.	n.d.	n.d.
Histidine	n.d.	n.d.	n.d.	n.d.
Arginine	n.d.	n.d.	n.d.	n.d.
Tyrosine	n.d.	n.d.	n.d.	n.d.
Cysteine	n.d.	n.d.	n.d.	n.d.

Supplementary Table S3: Amino acid formation rate rp, specific amino acid formation rate qp and the amino acid yields determined via LC-ESI-MS analysis of the supernatant of condition “High”. Measurement was performed for 15 amino acids; n.d. indicates that the concentration for the amino acid was below the limit of quantification of the analytical method. The notation n.d. stands for “not determined” due to an amino acid concentration below the limit of quantification (see Supplementary Table S1).

	r_p [g_p/h]	q_p [g_p/g_x/h]	$Y_{p/s}$ [g_p/g_s]	$Y_{p/s}$ [Cmol p/Cmol s]
Glycine	8.99E-04	4.54E-05	3.83E-04	3.82E-05
Alanine	3.08E-05	1.56E-06	1.31E-05	1.43E-05
Proline	2.22E-04	1.12E-05	9.46E-05	1.33E-04
Valine	7.88E-04	3.98E-05	3.35E-04	4.63E-04
Threonine	7.58E-05	3.83E-06	3.22E-05	3.51E-05
Isoleucine	2.37E-05	1.20E-06	1.01E-05	1.50E-05
Aspartate	7.58E-05	3.83E-06	3.22E-05	3.02E-05
Lysine	6.57E-06	3.32E-07	2.79E-06	3.58E-06
Phenylalanine	n.d.	n.d.	n.d.	n.d.
Leucine	n.d.	n.d.	n.d.	n.d.
Methionine	n.d.	n.d.	n.d.	n.d.
Histidine	n.d.	n.d.	n.d.	n.d.
Arginine	n.d.	n.d.	n.d.	n.d.
Tyrosine	n.d.	n.d.	n.d.	n.d.
Cysteine	n.d.	n.d.	n.d.	n.d.

Supplementary Table S4: Amino acid formation rate rp, specific amino acid formation rate qp and the amino acid yields determined via LC-ESI-MS analysis of the supernatant of condition "Overfeed". Measurement was performed for 15 amino acids; n.d. indicates that the concentration for the amino acid was below the limit of quantification of the analytical method. The notation n.d. stands for "not determined" due to an amino acid concentration below the limit of quantification (see Supplementary Table S1).

	rp [gp/h]	qp [gp/gx/h]	Yp/s [gp/gs]	Yp/s [Cmol p/Cmol s]
Glycine	9.00E-03	4.52E-04	2.76E-03	3.89E-04
Alanine	5.97E-03	3.00E-04	1.83E-03	1.99E-03
Proline	2.91E-04	1.46E-05	8.92E-05	1.25E-04
Valine	6.76E-02	3.40E-03	2.07E-02	2.85E-02
Threonine	6.10E-04	3.06E-05	1.87E-04	2.03E-04
Isoleucine	1.34E-03	6.72E-05	4.10E-04	6.06E-04
Aspartate	3.42E-04	1.72E-05	1.05E-04	9.83E-05
Lysine	2.17E-05	1.09E-06	6.64E-06	8.52E-06
Phenylalanine	6.13E-05	3.08E-06	1.88E-05	3.31E-05
Leucine	n.d.	n.d.	n.d.	n.d.
Methionine	n.d.	n.d.	n.d.	n.d.
Histidine	n.d.	n.d.	n.d.	n.d.
Arginine	n.d.	n.d.	n.d.	n.d.
Tyrosine	n.d.	n.d.	n.d.	n.d.
Cysteine	n.d.	n.d.	n.d.	n.d.

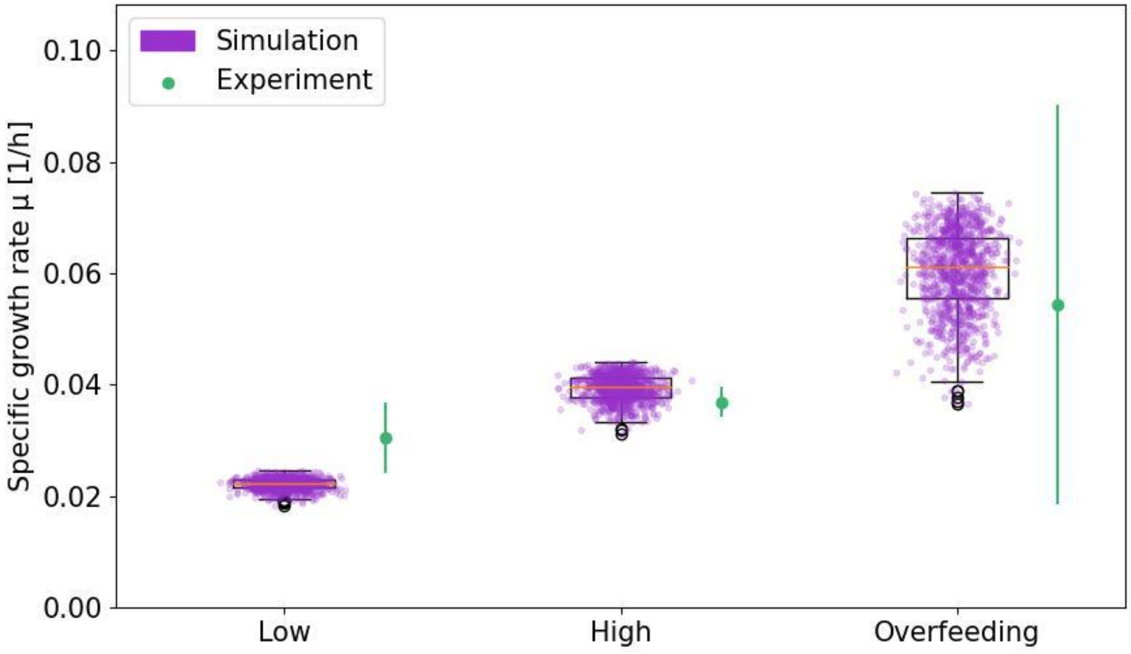

Supplementary Figure S1. Comparison of the experimental specific growth rate and specific growth rate acquired by the simulation using a published model for Saccharolobus solfataricus P2 (Wolf et al., 2016) for the conditions “Low”, “High” and “Overfeed”.

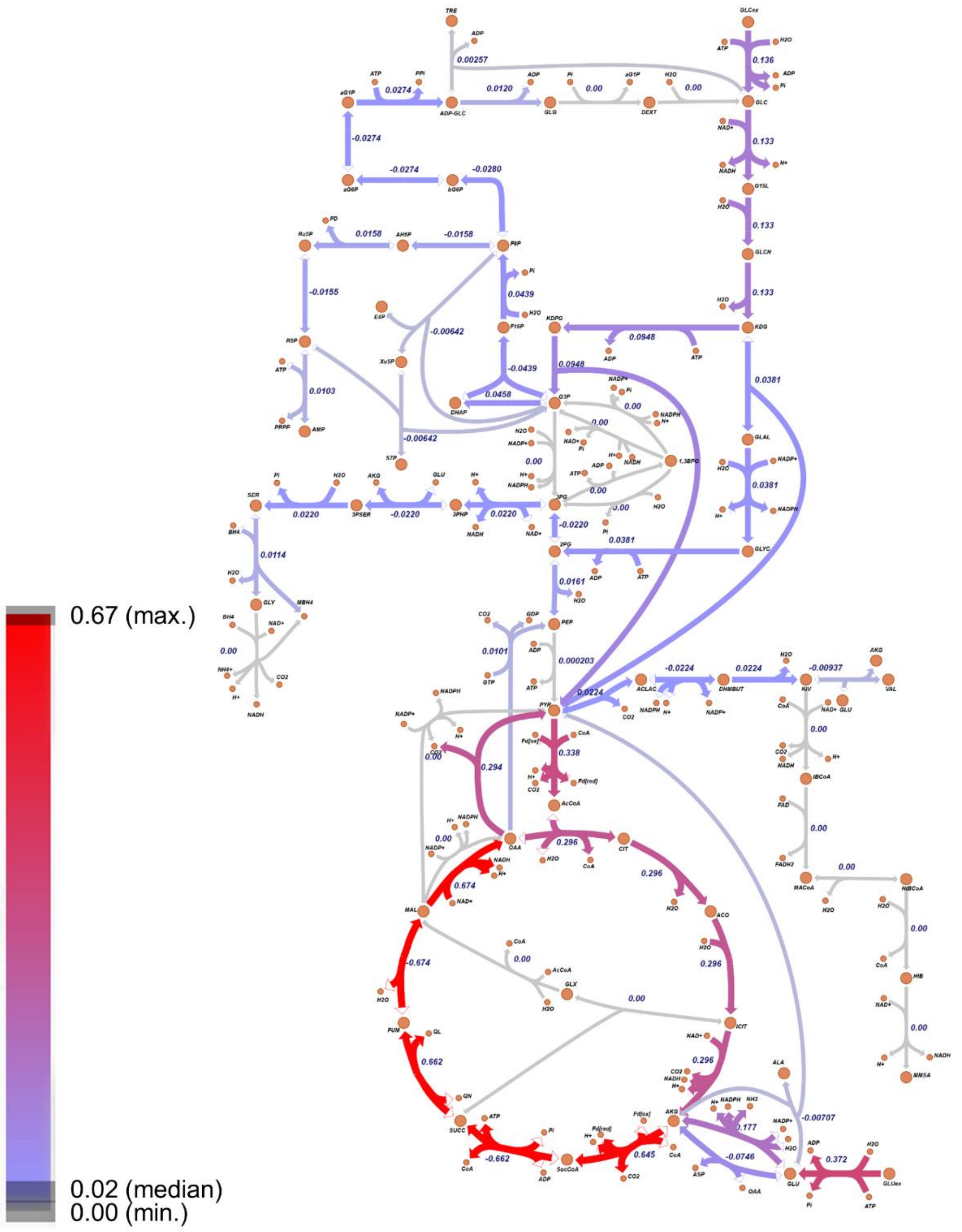

Supplementary Figure S2. Detailed results of parsimonious flux balance analysis for pathways of the central carbon metabolism of *S. acidocaldarius* cultivated in the condition “Low”

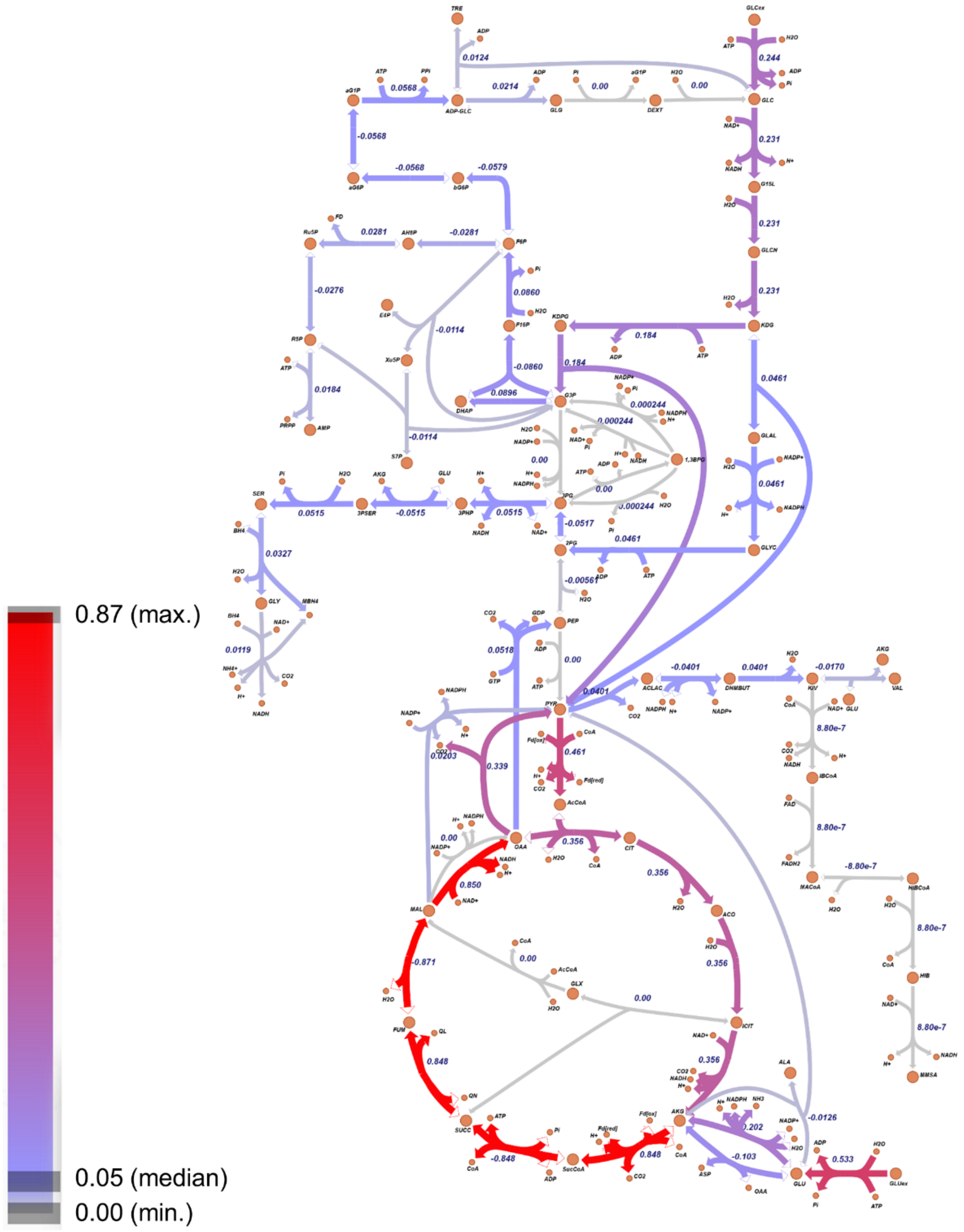

Supplementary Figure S3. Detailed results of parsimonious flux balance analysis for pathways of the central carbon metabolism of *S. acidocaldarius* cultivated in the condition “High”.

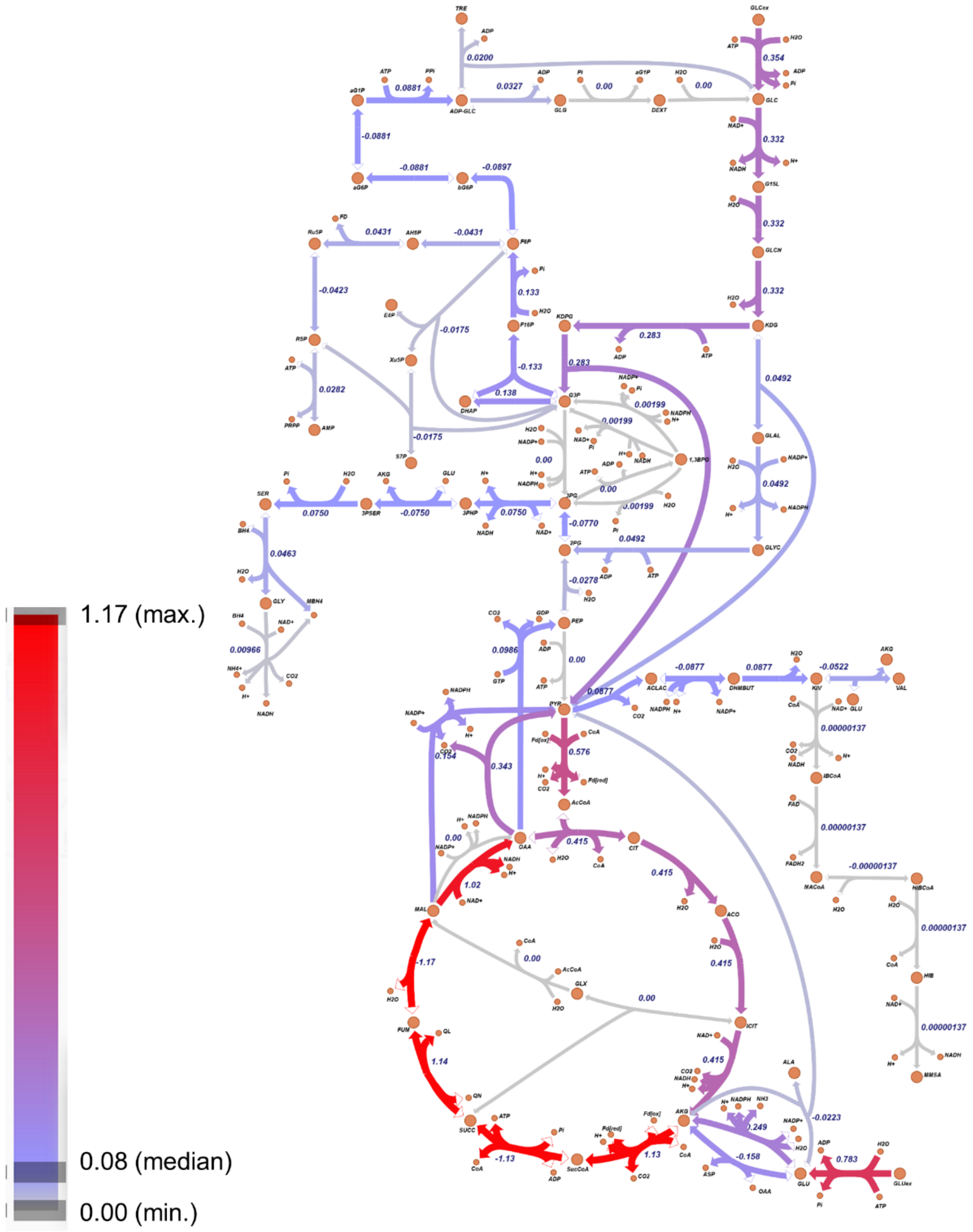

Supplementary Figure S4. Detailed results of parsimonious flux balance analysis for pathways of the central carbon metabolism of *S. acidocaldarius* cultivated in the condition “Overfeed”.
